# Circulating microRNAs Are Associated With Metabolic Markers in Adolescents With Hepatosteatosis

**DOI:** 10.3389/fendo.2022.856973

**Published:** 2022-04-14

**Authors:** Haixia Lin, Kelly E. Mercer, Xiawei Ou, Kori Mansfield, Robert Buchmann, Elisabet Børsheim, Emir Tas

**Affiliations:** ^1^ Arkansas Children’s Nutrition Center, Little Rock, AR, United States; ^2^ Department of Pediatrics, University of Arkansas for Medical Sciences, Little Rock, AR, United States; ^3^ Center for Childhood Obesity and Prevention, Arkansas Children’s Research Institute, Little Rock, AR, United States; ^4^ Department of Radiology, University of Arkansas for Medical Sciences, Little Rock, AR, United States; ^5^ Endocrinology and Diabetes, Arkansas Children’s Hospital, Little Rock, AR, United States

**Keywords:** childhood obesity, insulin resistance, liver disease, MRI, microRNA

## Abstract

**Background:**

Altered hepatic microRNA (miRNA) expression may play a role in the development of insulin resistance (IR) and non-alcoholic fatty liver disease (NAFLD). Circulating miRNAs could mirror the liver metabolism.

**Objective:**

This study aimed to assess the relationship between serum miRNA profile in children with obesity, IR, and NAFLD.

**Methods:**

Adolescents with obesity (n = 31) were stratified based on insulin resistance and NAFLD status. One-hundred seventy-nine miRNAs were determined in the serum by quantitative RT-PCR. Differentially expressed miRNAs were compared between groups, and log-transformed levels correlated with metabolic markers and intrahepatic triglyceride.

**Results:**

Serum miR-21-5p, -22-3p, -150-5p, and -155-5p levels were higher in children with IR and NAFLD, and their expression levels correlated with hepatic fat and serum triglyceride. In patients with NAFLD, miR-155-5p correlated with ALT (r = 0.68, p<0.01) and AST (r = 0.64, p<0.01) and miR-21-5p and -22-3p levels correlated with plasma adiponectin (r = -0.71 and r = -0.75, respectively, p<0.05) and fibroblast growth factor-21 (r = -0.73 and r = -0.89, respectively, p<0.01). miR-27-3a level was higher in children without IR and NAFLD.

**Conclusions:**

Several miRNAs are differentially expressed in children with IR and NAFLD. Determining their mechanistic roles may provide newer diagnostic tools and therapeutic targets for pediatric NAFLD.

## Introduction

Non-alcoholic fatty liver disease (NAFLD) encompasses a broad spectrum of liver diseases ranging from simple steatosis to non-alcoholic steatohepatitis (NASH) with or without fibrosis (1). NAFLD is the most common cause of chronic liver disease in children and is believed to follow a more aggressive course compared to adult disease due to early-onset and distinct histological features. Yet, the prevalence of pediatric NAFLD has been difficult to assess clinically and there is no approved pharmacotherapy for its treatment.

The etiopathogenesis of NAFLD in children is complex. It is regarded as the hepatic manifestation of the metabolic syndrome given its strong association with insulin resistance (IR), type 2 diabetes (T2D), and dyslipidemia, but is also an independent risk factor for cardiovascular morbidity and mortality. Early diagnosis through screening followed by treating associated comorbidities, i.e., obesity and IR, is the standard of care. However, the commonly available screening tests have major limitations, for example serum ALT has low specificity and liver ultrasonography has low sensitivity, whereas reference standards such as liver biopsy and MRI are expensive and not readily accessible in most centers (2). Additional research is needed to identify molecular mechanisms and/or novel biomarkers to improve diagnostic accuracy and provide potential targets for pharmacotherapy. Recent research has highlighted the role of epigenetic factors on NAFLD development. MicroRNAs (miRNAs), a type of short non-coding RNAs in the length of 19-28 nucleotides, are implicated in the epigenetic regulation of gene expressions involved in the pathogenesis of NAFLD (3). Dysregulation of hepatic miRNAs is associated with NAFLD (4–6). For instance, decreased hepatic miR-122 promotes hepatic *de novo* lipogenesis, which is implicated in the development of steatosis and progression to NASH (7); circulating miR-122 and miR-192 may distinguish NAFLD patients from healthy controls; and miRNA-34a may differentiate NASH from steatosis (8). Interestingly, one clinical cohort demonstrated that the classification performance of validated miRNAs (or their ratios) for NASH is better than that reached by ALT or aspartate aminotransferase (AST) (9). In addition, cross-validated models combining both clinical and miRNA variables showed an enhanced prediction of NAFLD. Taken together these findings show that circulating miRNAs correlate to the molecular events contributing to the pathogenesis of NAFLD and monitoring miRNAs may improve the accuracy of diagnostic screening tools.

Pediatric studies investigating the relationship between miRNA expressions and clinical and metabolic markers in children at-risk for NAFLD are limited. Most studies utilized healthy children without obesity as controls (6, 10–12) and one study (5) compared miRNA profile among pre-pubertal children. Considering the puberty-associated IR and strong association between onset of puberty and NAFLD, a more appropriate control group, one with similar risk factors is needed to decipher the effect of obesity or associated metabolic complications such as IR on miRNA profile in patients with NAFLD. In the present study, we aim to determine the associations between circulating miRNAs and metabolic and hepatic features of NAFLD and serum levels of insulin, adiponectin, and fibroblast growth factor (FGF)-21 in children with obesity and varying degrees of IR and intrahepatic triglyceride (IHTG).

## Materials and Methods

### Study Design and Subject Recruitment

This miRNA expression study was a secondary analysis of data collected to examine the role of FGF21 in NAFLD and prediction of changes in intrahepatic triglyceride (IHTG) percent in children with obesity presenting an outpatient weight management clinic during a 6-month observational study (13). The Institutional Review Board of the University of Arkansas for Medical Sciences approved the study. Parental consent and participant assent from all participants < 18 years old were obtained as previously described. Briefly, sixty-one pubertal children (aged 10-17 years, Tanner stage II and up) with a body mass index (BMI) ≥ 95^th^ percentile for age and sex, with no underlying medical problems including diabetes and liver diseases, were randomly recruited. Fasting serum samples were collected, and liver magnetic resonance imaging (MRI) was performed in all participants at baseline and 6 months later. Forty-nine children completed the study at 6 months. Serum miRNA expressions were determined in thirty-one children only at 6-month due to sample availability. As such, all-comparative analyses were performed on the data obtained at 6 months.

### Anthropometric Measurements, Body Composition and Clinical Analyte Detection

Anthropometric measurements, including weight, height, BMI, age, and sex-adjusted BMI percentile, were collected. The clinical analytes, including serum concentrations of glucose, insulin, triglycerides (TG), ALT, AST, gamma-glutamyl transferase (GGT), total cholesterol (TC), high-density lipoprotein cholesterol (HDL-C), and low-density lipoprotein cholesterol (LDL-C) were determined using a clinical analyzer (Siemens Atellica, Malvern, PA, USA) at the Arkansas Children’s Hospital Chemistry Laboratory. Fasting free fatty acids (FFA) were measured *via* a chemistry analyzer (Randox Daytona, Holliston, MA, USA) in the Metabolism and Bioenergetics Core at the Arkansas Children’s Research Institute (ACRI). Fasting serum leptin (Human Leptin Quantikine ELISA), adiponectin (Human Total Adiponectin/Acrp30 Quantikine ELISA), and FGF21 (Human FGF21 Quantikine ELISA) were measured according to manufacturer’s instructions (R&D Systems, Minneapolis, MN, USA) in the Metabolism and Bioenergetics Core at the ACRI. Insulin resistance was estimated using the Homeostatic Model Assessment of Insulin Resistance (HOMA-IR), calculated using the formula insulin (µIU/mL) × glucose (mg/dL)/405. A HOMA score of 4 was used to classify subjects with IR (HOMA-IR > 4), given that the risk of developing Type 2 Diabetes is relatively low below this HOMA-IR level (14).

### Quantification of Intrahepatic Triglyceride

We estimated IHTG percent using magnetic resonance imaging (MRI) as previously described (13). In brief, a multi-echo multi-slice gradient-echo pulse sequence with TR 150 ms, flip angle 25 degrees, and echo times of 2.3 ms, 4.6 ms, and 9.2 ms with breath-hold were used to acquire in/out of phase images of the whole liver using a 1.5T MRI scanner (Philips Healthcare, Best, The Netherlands). The confounding effects of intrinsic T2/T1 relaxation in the liver fat quantification were controlled by the triple-echo method. Raw MRI images were downloaded to a workstation with MATLAB software (The MathWorks Inc., Natick, MA, USA) and customized scripts for data analysis. Two raters first sketched a region-of-interests (ROI) for each subject which included the whole liver as much as possible but avoided intrahepatic vessels and perihepatic fat as well at all edges. The average signal intensity in the selected ROI for each echo time was computed, and the liver fat concentration for the subject was calculated from these signal intensities as described (13). Participants were diagnosed with NAFLD *via* MRI if liver fat content was ≥ 5%. Raters were blinded to the anthropometric and biochemical data of the subjects to avoid interpretation bias in the MRI data analysis.

### Serum RNA Extraction and miRNA Profiling

Total RNA, including microRNA, in 200 uL serum were extracted using miRNeasy Serum/Plasma Kit (Qiagen, Valencia, CA, USA) per the manufacturer’s instruction. One microliter of RNA (total 14 uL) was reverse transcribed using the miRCURY LNA RT Kit (Qiagen). miRNA was amplified with 179 different primers on the Human Serum/Plasma miRCURY LNA miRNA Focus PCR panels (96-well format panel I& II, YAHS-106Y; Qiagen) using locked nucleic acids (LNA) technology and miRCURY LNA SYBR Green PCR kit (Qiagen). Spike-in controls: *C.elegans* miRNA (cel-miR-39-3p), UniSp6, UniSp2, UniSp4, and Unisp5 were added to each plate for monitoring RNA isolation, cDNA synthesis, and PCR amplification. Sample quality and hemolysis were assessed using miScript PCR Controls (Qiagen). The miRNA profiles were performed using a quantitative RT-PCR (qRT-PCR) on a Fast 7500 Real-time PCR System, Applied Biosystems (Life Technologies, Foster City, CA, USA). Amplicons were analyzed for distinct melting curves, and the T_m_ was checked to the within known specifications for the assays. qRT-PCR data were analyzed using the ∆Ct method and normalized to a normalization factor calculated based on GeNorm methodology from the entire panel (15). All the miRNAs were assessed for the least variance across all samples in groups. According to the GeNorm analysis, selecting miR-486-5p, -193-5p, -101-3p, and let-7a-5p as normalizers showed the least variance and was confirmed by comparison of the five suggested spike-in controls. UniSp3 was used to do inter-plate calibration and correct for variances across plates. miRNA with Ct values > 35 in at least 65% of samples was excluded.

### Statistical Analysis

Data are presented as mean ± standard deviation except where otherwise indicated. Categorical proportions (e.g., sex and ethnicity) were determined by Fisher’s exact test. For multiple-group comparison, one-way ANOVA was conducted, followed by Tukey or Dunn all pairwise comparisons *post hoc* analysis to compare groups to each other. For two-group comparison, Student’s t-test was used for analytes that were normally distributed and a Mann-Whitney test for analytes not normally distributed (as defined by *p* < 0.05, determined by D’Agostino-Pearson normality test). Correlations between miRNA expression levels (-log2) and clinical and biological parameters (independent variables) were determined using Pearson’s correlation coefficients for normally distributed data or Spearman correlation coefficients for not normally distributed data. All statistical analyses were performed using GraphPad Prism7 (GraphPad Software, Inc., La Jolla, CA, USA). Significance was considered as *p* < 0.05.

## Results

### Characteristics of Participants

We have previously shown that IR in adolescents with obesity is associated with a specific miRNA signature (16). Therefore, given the wide-range of insulin concentrations and HOMA-IR levels in the non-NAFLD group, we stratified the non-NAFLD group into i) Non-NAFLD without IR (n = 7), and ii) Non-NAFLD with IR (n = 8) to explore the effects of IR and NAFLD on miRNA expression pattern in the circulation. The clinical and metabolic characteristics of participants based on IR and NAFLD status are summarized in **Table 1**. As per design, subjects in the NAFLD group (n = 16) had a higher mean hepatic fat percentage than the groups without NAFLD (median IHTG 10.43%, range 5.04 – 23.09%). In addition, all subjects in the NAFLD group had IR (i.e., HOMA-IR > 4). The groups were similar regarding sex distribution, mean age, BMI, and BMI percentile (*p* > 0.05 for all). Serum insulin and HOMA-IR levels were higher in the NAFLD group (*p* < 0.05 for all). Other metabolic markers including serum glucose, cholesterol (Total, HDL, LDL), FFA, liver enzymes (ALT, AST, GGT), FGF21, adiponectin, and leptin were not different between the groups.

**Table 1:** Characteristics of participants according to insulin resistance and NAFLD status.

	Non-NAFLD		NAFLD
	without IR (n=7)	with IR (n=8)	(n=16)
Age (years)	14 ± 1.4	14.1 ± 2.4	14.1 ± 2.1
Sex (Male : Female)	4:3	4:4	8:8
BMI (kg/m^2^)	35.7 ± 5.5	36.2 ± 4.7	37.1 ± 4.5
BMI percentile	99 ± 0.64	98.9 ± 0.62	99.5 ± 1.04
IHTG (%)	2.9 ± 1.3	3.4 ± 1.1	13.1 ± 7^a, b^
Glucose (mg/dL)	88 ± 11	94 ± 9	93 ± 10
Insulin (µIU/mL)	15.2 ± 3.1	27.6 ± 7.5^a^	52.2 ± 33.3^a, b^
HOMA-IR	3.3 ± 0.7	6.3 ± 1.7^a^	12.4 ± 8.5^a, b^
Leptin (pg/mL)	42 ± 29	56 ± 20	51 ± 24
Adiponectin (ng/mL)	6.5 ± 3.8	7.4 ± 2.9	8.2 ± 5.2
FGF21 (pg/mL)	181 ± 164	202 ± 136	242 ± 174
Triglyceride (mg/dL)	67 ± 27	126 ± 74	169 ± 61^a^
Total Chol (mg/dL)	133 ± 10	165 ± 40	152 ± 24
HDL-Chol (mg/dL)	44 ± 6	40 ± 7	37 ± 7
LDL-Chol (mg/dL)	75 ± 7	101 ± 42	80 ± 21
ALT (IU/L)	34 ± 10	34 ± 22	47 ± 29
AST (IU/L)	26 ± 4	27 ± 8	33 ± 13
GGT (IU/L)	27 ± 10	20 ± 8	29 ± 13
FFA (mmol/L)	4.6 ± 1.9	4.3 ± 1.6	4.2 ± 1.7

Data are expressed as mean ± standard deviation (SD). Significant differences between groups were determined by one-way ANOVA followed by post-hoc all-pairwise comparison. Labeled (a or b) means difference for comparison of two groups following the ANOVA analysis. ALT, alanine aminotransferase; AST, aspartate aminotransferase; FFA, free fatty acids; FGF, fibroblast growth factor; GGT, gamma-glutamyl transferase; IHTG, intrahepatic triglyceride. ^a^p < 0.05 compared with non-NAFLD without IR group; ^b^p < 0.05 compared with non-NAFLD with IR group.

### miRNA Expression Pattern

**Figure 1:**
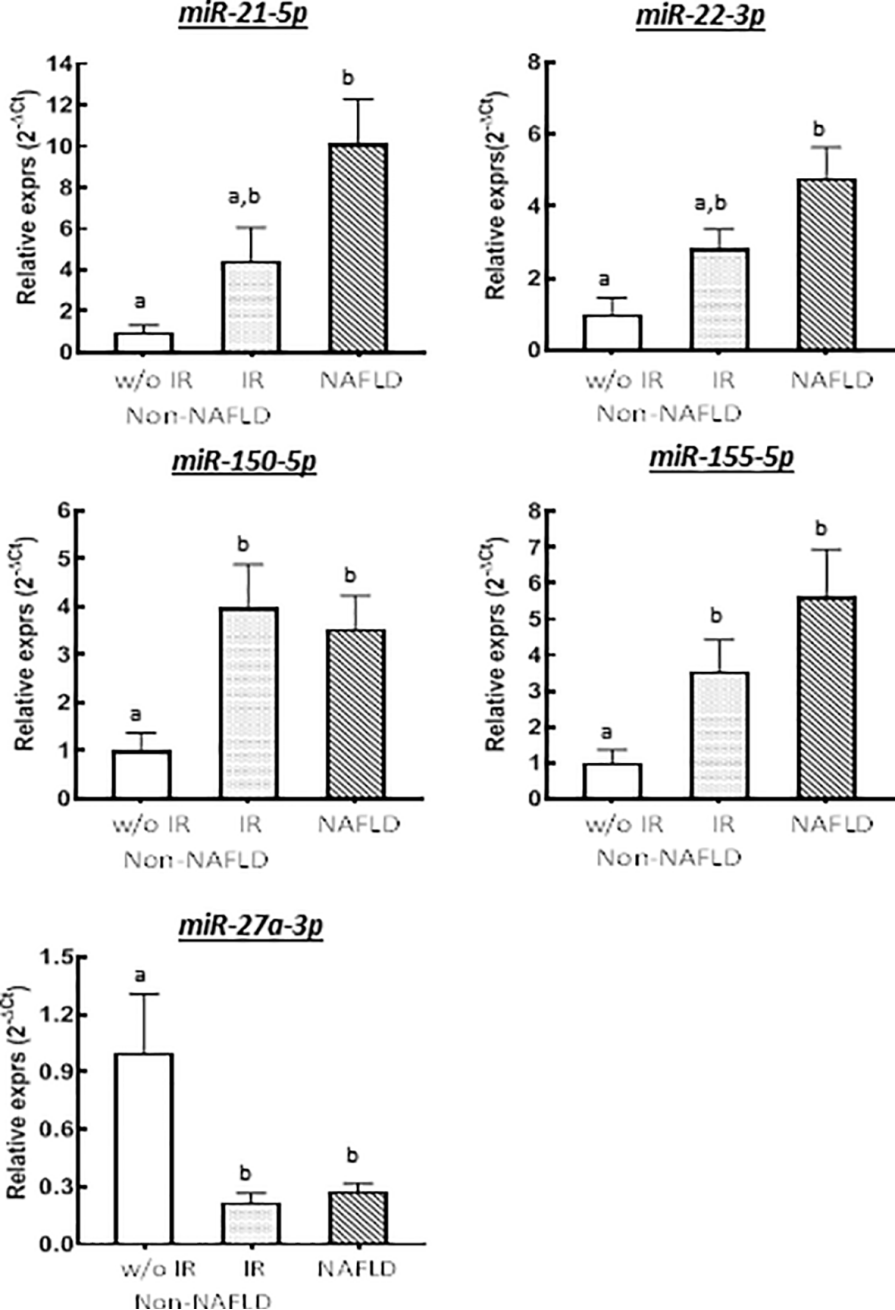
Relative expressions of differentially expressed miRNAs across groups stratified by IR and NAFLD status (n = 7 for Non-NAFLD without IR, n = 8 for Non-NAFLD with IR, and n = 15 for NAFLD groups). Group means were compared by one-way ANOVA. Labeled (a or b) means difference for comparison of two groups following the ANOVA analysis. Bars represent standard error of the means.

In miRNA profiling, we identified five miRNAs that were differentially expressed between the three groups (Non-NAFLD without IR, Non-NAFLD with IR, and NAFLD), including miR-21-5p, -22-3p, -150-5p, -155-5p, and -27a-3p (**Figure 1**).

Expression levels of miR-21 and -22 were significantly higher in subjects with NAFLD compared to non-NAFLD without IR group (10.2-fold [*p* = 0.011] and 3.6-fold [*p* = 0.038] increase, respectively) **(** **Figures 1A, B** **),** but they were not significantly higher compared to non-NAFLD with IR group (4.4-fold increase for miR-21, *p* = 0.142, and 4-fold increase for miR-22, *p* = 0.841). miR-150 and miR-155 were both higher in the NAFLD (3.5-fold [*p* = 0.030] and 5.6-fold [*p* = 0.034] increase, respectively) and non-NAFLD with IR (4.0-fold [*p* = 0.012] and 3.5- fold [*p* = 0.025] increase, respectively) groups compared to non-NAFLD without IR group (**Figures 1C, D** **)**. In contrast, miR-27a-3p expression was significantly higher in the non-NAFLD without IR group compared to non-NAFLD with IR (4.5-fold decrease, [*p*=0.002]) and NAFLD (3.7-fold decrease [*p* = 0.018]) groups (**Figure 1E**).

### Associations Between Differentially Expressed miRNAs and Metabolic Markers of IR and IHTG

The correlation analyses across three groups showed that expression profiles of five miRNAs (-21-5p, -22-3p, -150-5p, -155-5p, -27a-3p) were correlated with serum markers of IR and lipid metabolism, and hepatic enzyme levels in the circulation (**Table 2**). In the non-NAFLD without IR group, miR-150-5p was positively correlated with TC, miR-155-5p was negatively correlated with HDL-C, and miR-27a-3p was negatively correlated with TG. In the non-NAFLD with IR group, miR-150-5p was positively correlated with TC and LDL-C, miR-155-5p was positively correlated with insulin but negatively with adiponectin, miR-22-3p level was negatively correlated with adiponectin, and miR-27a-3p was negatively correlated with insulin and TG. In the NAFLD group, miR-150-5p was positively correlated with TG, miR-155-5p was positively correlated with insulin, TG, and TC, miR-21-5p and miR-22-3p were both positively correlated with TG and LDL-C and negatively correlated with adiponectin, and miR-27a-3p was negatively correlated with insulin and TG. In the NAFLD group only, while IHTG was positively correlated with miR-21-5p, -22-3p, -150-5p, and -155-5p, it was negatively correlated with miR-27a-3p expression. There was a negative correlation between FGF21 and miR-22-3p in the non-NAFLD with IR and NAFLD groups, and FGF21 and miR-21-5p only in the NAFLD group. Finally, ALT was negatively correlated with miR-27a-3p in the Non-NAFLD without IR group, and ALT and AST were both positively correlated with miR-155-5p in the NAFLD group (**Table 2**).

**Table 2:** Correlation analysis between serum miRNAs and metabolic markers among the groups.

Variables	Non-NAFLD without IR (n=7)					Non-NAFLD with IR (n=8)					NAFLD (n=16)				
	miR-21	miR-22	miR-150	miR-155	miR-27a	miR-21	miR-22	miR-150	miR-155	miR-27a	miR-21	miR-22	miR-150	miR-155	miR-27a
	-5p	-3p	-5p	-5p	-3p	-5p	-3p	-5p	-5p	-3p	-5p	-3p	-5p	-5p	-3p
	r	r	r	r	r	r	r	r	r	r	r	r	r	r	r
Insulin (µIU/mL)	0.26	0.36	0.53	-0.46	0.36	-0.19	-0.10	-0.39	0.82*	-0.78*	-0.34	-0.14	0.23	0.73**	-0.74*
FGF21 (pg/mL)	-0.43	-0.23	-0.22	0.31	-0.13	-0.54	-0.74*	-0.54	0.60	0.48	-0.73**	-0.89**	0.18	0.43	0.52
Adiponectin (ng/mL)	0.39	0.19	-0.38	0.71	-0.09	-0.53	-0.79*	0.51	-0.86**	0.32	-0.71*	-0.75*	-0.15	-0.82**	0.22
IHTG (%)	0.21	0.34	0.64	0.34	-0.32	0.42	0.34	-0.35	-0.01	-0.56	0.75**	0.78**	0.62**	0.64*	-0.68*
Triglyceride (mg/dL)	0.41	0.46	0.12	0.21	-0.86**	0.54	0.34	-0.36	0.28	-0.78*	0.74*	0.85*	0.64*	0.69**	-0.72*
Total Chol (mg/dL)	0.52	0.22	0.82*	-0.66	0.21	0.38	0.18	0.80*	-0.51	-0.26	-0.51	-0.13	-0.05	0.54*	-0.14
HDL-Chol (mg/dL)	0.28	0.48	*0.63*	-0.87**	0.40	0.15	0.40	0.36	-0.31	0.54	-0.46	-0.26	0.50	-0.44	0.36
LDL-Chol (mg/dL)	0.14	0.34	0.58	-0.45	0.64	0.32	0.20	0.81**	-0.52	-0.36	0.68*	0.72*	-0.29	0.38	0.46
ALT (IU/L)	0.48	0.48	0.37	-0.08	-0.64*	0.42	0.46	0.04	-0.41	-0.46	0.35	0.35	-0.35	0.68**	-0.35
AST (IU/L)	-0.23	-0.53	0.07	-0.46	-0.53	0.32	0.47	-0.08	-0.39	-0.03	0.46	0.26	-0.26	0.64**	0.25

ALT, alanine aminotransferase; AST, aspartate aminotransferase; FGF, fibroblast growth factor; IHTG, intrahepatic triglyceride; IR, insulin resistance; NAFLD, Non-Alcoholic Fatty Liver Disease. miRNA levels were transformed into a log scale (-log2). r represents correlation coefficient, shown in bold italic when the p-value is less than 0.05. *represents p < 0.05; **represents p < 0.01.

## Discussion

Hepatic miRNAs are involved in lipid and glucose metabolic pathways; however, little is known about associations of miRNA expression in pediatric NAFLD. In this secondary analysis, we investigated the relationships between serum miRNA profile, metabolic biomarkers (insulin, lipid profile, adipokines, and liver enzymes), and IHTG percent in a well-described cohort of pubertal children with obesity and varying degrees of IR. We provided new evidence that serum concentrations of miR-21-5p, -22-3p, -150-5p, and -155-5p were higher in patients with NAFLD, and the metabolic profile of adolescents with IR were comparable among those with and without NAFLD. We also demonstrated that miR-27-3a expression was higher in adolescents without IR compared to those with IR regardless of NAFLD status.

A growing body of evidence suggests that the expression pattern of several miRNAs, including miR-21 and -22, are associated with the severity of NAFLD. miR-21 and -22 are two of the most abundantly expressed liver miRNAs. miR-21 is hypothesized to regulate the genes in hepatic cholesterol and triglyceride metabolisms (17). Furthermore, ablation of hepatic miR-21 was shown to decrease hepatic inflammation and improve fibrosis (18). In our study, we showed that serum miR-21 and -22 were both positively correlated with IHTG, TG, and LDL-C only in the NAFLD group. No correlation was observed between these miRNAs and liver enzymes in any of the groups. Based on these results it is tempting to speculate that in children with obesity and IR, increased expression of miR-21 and -22 in the circulation could be a harbinger of impending NAFLD. However, a limited number of studies in adults have reported contradictory results about miR-21 expression pattern. Sun et al. demonstrated decreased concentration of miR-21 in the circulation in patients with NAFLD (19), while Becker and colleagues did not find any difference between the control and NAFLD groups, but higher levels in patients with NASH (7). Moreover, while the role of miR-22 in the progression of steatosis to advanced stages such as fibrosis and cirrhosis is recognized (20), its role in the development of steatosis is less understood. Further validation studies are needed to determine if miR-21 and -22 are predictive markers for NAFLD progression in pediatric populations.

In the current study, we also showed that miR-22 expression negatively correlated with FGF21 and adiponectin levels in adolescents with IR regardless of NAFLD status. There were no differences in FGF21 levels among the groups with or without IR or NAFLD. We speculate that increased miR-22 blunts hepatic FGF21 synthesis and secretion in children with NAFLD, and therefore promotes worsening of steatosis. In a recent study, Hu et al. (21) utilizing a human liver cell line and fatty liver specimens demonstrated that increased hepatic miR-22 expression inhibited FGF21 expression and promoted lipogenesis through directly suppressing fibroblast growth factor receptor 1 (FGFR1) while reducing PPARα and PPARγ coactivator 1α (PGC1α), two transcriptional factors regulating FGF21 expression. Moreover, they also showed inhibition of miR-22 eliminates alcohol-induced steatosis in murine models, possibly due to restoring FGF21 expression. Considering the beneficial properties of FGF21 in hepatic lipid metabolism (22), these findings provide insight on the mechanistic role of miR-22 in NAFLD development.

Altered hepatic and serum levels of miR-150 have been reported in animal models and patients with insulin resistance and liver diseases. However, whether miR-150 exerts a protective role in NAFLD or promotes development and progression of the steatosis into more advanced stages is yet to be determined. Zhuge et al. (23) demonstrated elevated levels of miR-150 in the serum of adult patients with NAFLD compared to healthy controls, and in the liver of mice fed with high-fat diet compared to chow-fed mice. They also reported that miR-150 knock-out mice were protected from developing hepatic steatosis and insulin resistance even when fed high fat diet. In line with these findings, Huang et al. showed that increased miR-150 expression promotes steatosis in human fetal hepatocyte line (24). On the contrary, Ying et al., also utilizing miR-150 deficient mice, showed that miR-150 plays an important regulatory role in adipose tissue inflammation and that deficiency of miR-150 in mice is associated with severe systemic inflammation and insulin resistance (25). Our findings did not support any relationship between miR-150 and markers of insulin resistance such as insulin, adiponectin, or FGF21, but showed that miR-150 might be an important regulator in the cholesterol and triglyceride metabolisms.

The mechanistic role of miR-155 in hepatic lipid metabolism is less understood and debated. Earlier studies suggested a protective role for miR-155, through its downstream effector liver X receptor, for the development of steatosis (26). In line with these reports, Johnson et al. (27) suggested that miR-155 expression in white adipose tissue is pivotal to prevent progression of obesity-associated inflammatory response. However, Ying et al. (28) reported that macrophage-derived exosomal miR-155 obtained from the adipose tissue of obese mice could induce IR in lean mice *via* its target PPARγ. More recently, Bala et al. (29) showed that miR-155 knock-out mice displayed less steatosis and fibrosis compared to wild type mice. Studies in humans are scarce and contradictory. While Wang et al. (30) demonstrated decreased liver and serum miR-155 levels in adult patients with NAFLD, Zhou et al. (31) showed increased serum levels in children with NAFLD. In the current study, we found positive correlations between serum miR-155 level and insulin and adiponectin concentrations in children with IR regardless of NAFLD status, and IHTG and liver enzymes in children with NAFLD only. Taken together, these findings may suggest that elevated miR-155 may play a role in the development and progression of NAFLD through induction or exacerbation of IR.

miR-27 is another understudied and potentially overlooked miRNA in the pathophysiology of pediatric NAFLD. In an *in vitro* study, Ji and colleagues (32) showed that overexpression of miR-27a and -27b were influential in fat accumulation in hepatic stellate cells. This was supported by the findings of Singaravelu et al. (33) who demonstrated that overexpression of miR-27b results in larger and more abundant lipid droplets in hepatocytes infected with hepatitis C virus, likely due to inhibition of PPARα. Increased expression levels of miR-27b were also shown in humans with NAFLD compared to healthy controls (4). On the contrary, Zhang et al. (34) showed that miR-27a, through the inhibition of two important regulatory genes in the fatty acid synthesis and thereby decreasing the rate of *de novo* lipogenesis, decreases lipid accumulation in the hepatocytes. In line with this report, we found increased serum miR-27 levels in adolescents without IR regardless of the NAFLD status. We observed negative correlations between miR-27 and TG for all groups, miR-27 and insulin for groups with IR, and finally miR-27 and IHTG for groups with NAFLD. In contrast to adult studies, our comparison group was composed of children with similar degrees of obesity and body fat percent. Since IR is considered the first-hit in the development of NAFLD, we speculate miR-27 plays a protective role and prevents excessive hepatic lipid deposition *via* regulation of hepatic insulin signaling pathways.

In conclusion, our results add to the growing body of literature showing miRNAs are important regulators of systemic glucose and hepatic lipid metabolism in children with obesity and varying degrees of IR. These findings may have clinical implications. Particularly miR-21-5p, -22-3p, -150-5p, and -155-5p may be used to identify patients with IR at-risk for developing NAFLD, while miRNA-27-3a could be used to identify those who have a low risk of developing IR and hence NAFLD. We acknowledge that the small cohort may not equally distribute potential confounders such as race/ethnicity or pubertal developmental stages among the groups being compared. In addition, given the cross-sectional design, a cause-and-effect relationship cannot be established in this association study. However, our findings do address the current need for more accurate diagnostic tools for determining patients with IR at-risk for NAFLD. Further characterization of the mechanical roles of these miRNAs in the pathogenesis of IR and NAFLD may help develop targeted pharmacotherapies.

## Data Availability Statement

The data analyzed in this study is subject to the following licenses/restrictions: The data that support the findings of this study (all of the individual participant data collected during the study - after deidentification, statistical analysis plan, and analytic code) are available from the corresponding author immediately following publication with the researchers who provide a methodologically sound proposal. Requests to access these datasets should be directed to ET, etas@uams.edu.

## Ethics Statement

The studies involving human participants were reviewed and approved by Institutional Review Board of the University of Arkansas for Medical Sciences. Written informed consent to participate in this study was provided by the participants’ legal guardian/next of kin.

## Author Contributions

HL and ET designed the research. ET conducted the study. HL, KEM, XO, KM, RB, EB, and ET processed and interpreted the clinical and imaging data. HL and ET performed the statistical analyses. HL and ET wrote the manuscript. ET had primary responsibility for final content and edits. All authors read and approve the final manuscript.

## Funding

Research reported in this publication was supported by the National Institute of General Medical Sciences of the National Institutes of Health under Award Number 5P20GM109096. HL, EB, KEM, and ET were also supported by the United States Department of Agriculture/Agricultural Research Service (USDA-ARS Project 6026-51000-012-06S).

## Conflict of Interest

The authors declare that the research was conducted in the absence of any commercial or financial relationships that could be construed as a potential conflict of interest.

## Publisher’s Note

All claims expressed in this article are solely those of the authors and do not necessarily represent those of their affiliated organizations, or those of the publisher, the editors and the reviewers. Any product that may be evaluated in this article, or claim that may be made by its manufacturer, is not guaranteed or endorsed by the publisher.

## Abbreviations

ALT, alanine aminotransferase; AST, aspartate aminotransferases; BMI, body mass index; FFA, free fatty acids; FGF21, fibroblast growth factor 21; GGT, Gamma-Glutamyl Transferase; IHTG, intrahepatic triglycerides; IR, insulin resistance; MRI, magnetic resonance imaging; NAFLD, nonalcoholic fatty liver disease.
